# Pharyngeal–Cervical–Brachial variant of Guillian–Barre Syndrome in Children

**DOI:** 10.7759/cureus.6983

**Published:** 2020-02-13

**Authors:** Ravi R Pradhan, Sant K Yadav, Shreebodh K Yadav

**Affiliations:** 1 Internal Medicine, Tribhuvan University Institute of Medicine, Kathmandu, NPL; 2 Internal Medicine, Provincial Hospital, Janakpurdham, NPL; 3 Internal Medicine, Scheer Memorial Adventist Hospital, Banepa, NPL

**Keywords:** gullian-barre syndrome, pharyngeal-cervical-brachial variant, atypical presentation, children, case report

## Abstract

Guillian-Barre Syndrome (GBS) typically presents as symmetrical ascending flaccid muscle weakness with areflexia, and with or without sensory symptoms. However, some patients may present atypically, and accordingly, different variants of GBS have been reported in the literature. Pharyngeal-cervical-brachial variant is one of the rare variants and is characterized by muscle weakness extending from the oropharyngeal and neck area to the proximal upper extremities. Many physicians and neurologists are unfamiliar about pharyngeal-cervical-brachial variant, which is often misdiagnosed as brainstem stroke, myasthenia gravis or botulism. Herein, we report a case of pharyngeal-cervical-brachial variant of GBS. To the best of our knowledge, this is the first reported case of pharyngeal-cervical-brachial variant of GBS in children from Nepal.

A 14-year-old Asian male presented with weakness of bilateral upper limb, dysphagia, and nasal intonation of voice. A diagnosis of pharyngeal-cervical-brachial variant of GBS was made after excluding all other possible differentials and based on cerebrospinal fluid analysis and nerve conduction study. The patient improved following conservative management.

Pharyngeal-cervical-brachial variant of GBS should always be considered in any patient presenting with symmetrical upper limb weakness and bulbar palsy. This is to ensure early diagnosis, treatment, and follow-up of the potential complications.

## Introduction

Guillian-Barre Syndrome (GBS) is an immune-mediated, inflammatory, demyelinating, polyradiculopathy, typically characterized by acute-onset symmetrical flaccid muscle weakness with decreased or absent deep tendon reflexes [1]. Patients usually present with an ascending paralysis that may be first noticed as rubbery legs associated with a tingling sensation in the lower extremities [2]. The lower cranial nerves are also frequently involved [3]. Several clinical reports have revealed the different variants of GBS. Pharyngeal-cervical-brachial (PCB) variant of GBS is characterized by muscle weakness involving oropharyngeal, neck, and upper extremity muscles, and was first reported by Ropper in 1986 [4-5]. Many physicians and neurologists are unfamiliar about PCB variant of GBS, which is often misdiagnosed as brainstem stroke, myasthenia gravis or botulism. The additional features of ophthalmoplegia and ataxia point towards overlap with Miller-Fisher syndrome [6]. Herein, we report a case of PCB variant of GBS. To the best of our knowledge, this is the first reported case of PCB variant of GBS in children from Nepal.

## Case presentation

A 14-year-old Asian male presented to our hospital with two weeks history of weakness of bilateral upper limbs, nasal intonation of voice, difficulty in swallowing and walking. Patient was in his usual state of health two weeks back when he experienced upper limb weakness, which was insidious in onset, gradually progressing for the first week, and static then after; associated with tingling sensations and numbness. He noticed that he had difficulty holding objects and with overhead movements. Seven days prior to admission, he noticed difficulty in swallowing, and his voice slowly became nasal, following which he also experienced difficulty in walking and clumsiness in lower limbs. There was no history of altered sensorium, loss of consciousness, and seizure. There was no bowel and bladder involvement at the onset of the disease. He didn’t experience any antecedent symptoms of upper respiratory tract infections or diarrhea. The patient’s previous medical history was unremarkable. He had not received any immunizations in the month prior to this incident. There was no history of drugs or heavy metal exposure. There was no similar history in other family members.

General physical examination was unremarkable. Patient had dysarthria, nasal intonation of voice with weak gag reflex suggestive of bulbar palsy. Uvula was central with decreased motion. The power of neck muscle was preserved, and there was no facial deviation. All other cranial nerves were intact. Power was of grade 4/5 in the upper limbs and 5/5 in the lower limbs in Medical Research Council (MRC) grading. Deep tendon reflexes were absent in bilateral upper and lower limbs, and planters were downgoing bilaterally. Sensations were preserved in bilateral upper and lower limbs. His single breath counting was normal. Examinations of other systems were unremarkable. Differential diagnosis of intracranial mass occupying lesion, myasthenia gravis, botulism, diphtheric polyneuropathy, brainstem stroke, and PCB variant of GBS were made.

His routine blood and urine investigations were within normal limits. Cerebrospinal fluid (CSF) examination revealed total leukocyte count, 5 cells/mm3; protein, 60 mg/dL; glucose, 3.5 mmol/L [Table 1].

**Table 1:** Laboratory parameters on admission

Parameters	Reference range, Children	On admission
White-cell count (per mm^3^)	4000-12000	6700
Diffferential count (%)		
Neutrophils	40-70	70
Lymphocytes	22-44	27
Eosinophils	0-10	2
Monocytes	4-11	1
Hemoglobin (gm/dl)	12-16	13.5
Random blood sugar (mmol/l)	3.5-7.0	3.8
Urea (mmol/l)	1.6-7.0	4.8
Creatinine (mmol/l)	60-115	90
Sodium (mEq/l)	135-145	137
Potassium (mEq/l)	3.5-5.2	4.3
Calcium (mmol/l)	2.1-2.6	2.3
Cerebrospinal fluid analysis		
White-cell count	0-5 (all lymphocytes)	5 (all lymphocytes)
Glucose (mmol/l)	2.3-4.6	3.1
Protein (mg/dL)	15-45	60
Red blood cell (per mm^3^)	0-0	0

**Figure 1:**
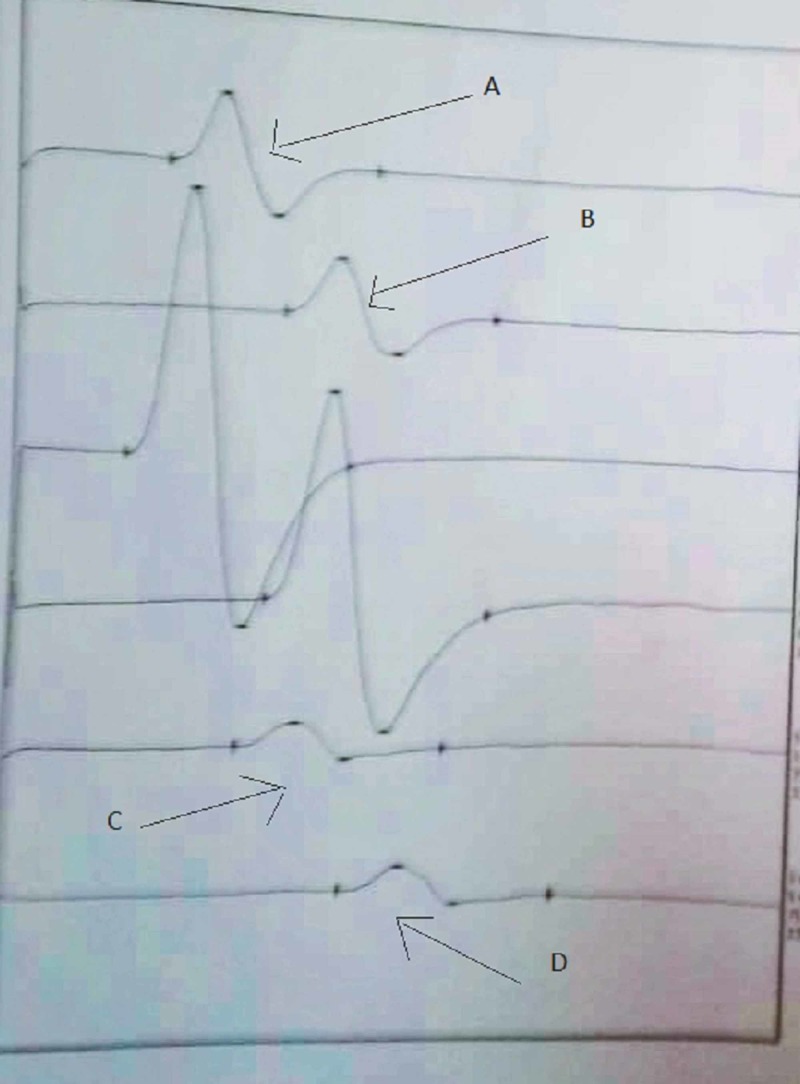
Nerve conduction study of motor nerves A and B: Increased distal and proximal latencies of right common peroneal nerve C and D: Increased distal and proximal latencies of left common peroneal nerve

Magnetic resonance imaging (MRI) of the brain and cervical spine were normal. Nerve conduction study (NCS) showed increased latencies of the right and left common peroneal nerves (CPN) (8.44 milliseconds and 13.5 milliseconds, respectively, normal: less than 6.5 milliseconds); features suggestive of acute inflammatory demyelinating polyneuropathy of motor nerves [Figure 1]. NCS of other nerves were normal. Antibody testing was not feasible due to financial constrains of the patient. A diagnosis of PCB variant of GBS was formulated based on clinical presentation, CSF findings, and NCS report.

He was managed conservatively as his symptoms were static at presentation to our center. At the time of discharge, gag reflex was present partially; power was 5/5 in bilateral upper and lower limbs. However, deep tendon reflexes were absent in bilateral upper and lower limbs. On follow-up visit after four weeks, the deep tendon reflexes also recovered.

## Discussion

The cardinal symptoms in our patient were the weakness of bilateral upper limbs, dysphagia, and nasal intonation of voice. First, we thought possibility of intracranial mass occupying lesion however, there were no signs of raised intracranial pressure, and brain imaging was also normal. Similarly, there was no history of sore throat, bull’s neck, and bleeding pharyngeal membrane therefore, the possibility of diphtheritic polyneuropathy was ruled out. There was no prior history of intake of honey, iridoplegia or autonomic dysfunction so, botulism was excluded. Based on clinical, laboratory, and electrophysiological findings of our patient, a diagnosis of GBS was formulated. Besides, our patient fulfilled the criteria for PCB variant of GBS [Table 2] [6].

**Table 2:** Diagnostic criteria for the pharyngeal–cervical–brachial variant of Guillain–Barre syndrome

Features required for diagnosis	Features strongly supportive of the diagnosis
1. Relatively symmetric oropharyngeal weakness and neck weakness and arm weakness and arm areflexia/ hyporeflexia	1. Antecedent infectious symptoms
2. Absence of ataxia and disturbed consciousness and prominent leg weakness	2. Cerebrospinal fluid albuminocytological dissociation
3. Monophasic illness pattern and interval between onset and nadir of oropharyngeal or arm weakness between 12 hours and 28 days and subsequent clinical plateau	3. Neurophysiological evidence of neuropathy
4. Absence of identified alternative diagnosis	4. Presence of IgG anti-GT1a or anti-GQ1b antibodies

Frequent facial muscle involvement has been reported in the patient with a lower cranial nerve form of GBS [3]. However, our patient didn’t have facial weakness. The pathogenesis involves molecular mimicry between gangliosides and microbial lipo-oligosaccharides [6]. History of antecedent upper respiratory tract infection was frequent than diarrhea. However, serological studies demonstrated that *Campylobacter jejuni* is the most common antecedent infection [6]. Our patient lacks the history of antecedent illness. In patients with PCB variant of GBS, the reflexes may only be absent in the arms, or there may be generalized areflexia [6]. The lower extremities are spared or mildly affected [6]. In our patient, power in the lower limbs was preserved (i.e. 5/5) however, there was generalized areflexia.

Patients are usually managed with intravenous immunoglobulins (IVIg) or plasmapheresis. However, our patient recovered rapidly after 10 days of admission, and no pharmacological intervention was instituted. Power of limbs also improved fast, and respiratory muscle was not compromised. Nasogastric tube insertion was planned but, the patient was able to swallow without regurgitating so, it was not inserted. This can be regarded as milder manifestation of the same immunopathogenetic process of PCB variant of GBS. A similar case of a 19-year-old female from Malaysia was reported who recovered within a week without any pharmacological intervention [7]. Both the patients of Bonanni et al. had unfavorable outcome and they died [8]. 

In the original report of Ropper, one patient had mild slowing of median nerve motor conduction velocity, and absent sensory action potentials in the arms, whereas another patient had absolutely normal NCS [5]. In the present patient, NCS showed increased distal latency of the right and left CPN and left PTN. PCB is regarded as a continuous spectrum of Miller-Fisher syndrome, and represents a localized form of axonal GBS based on clinical, immunological, and neurophysiological studies [6]. However, our patient didn’t complain of features suggestive of Miller-Fisher syndrome i.e. ataxia and ophthalmoplegia. Studies have shown that anti-GT 1a immunoglobulin G antibodies were positive in 51% of patients of PCB variant of GBS, whereas, anti-GQ1b immunoglobulin may or may not be present [9]. However, we were not able to test these immunoglobulins due to financial constraints of the patient.

## Conclusions

GBS may present atypically as PCB variant and likely to get misdiagnosed. So, one should have high index of suspicion for PCB variant in any patient presenting with symmetrical upper limbs weakness and bulbar palsy. Before confirming a diagnosis of PBC variant, one should rule out other differentials like: brainstem lesion, neuromuscular disorder, diphtheritic polyneuropathy, and botulism.
